# Pathophysiology of Type 2 Diabetes Mellitus

**DOI:** 10.3390/ijms21176275

**Published:** 2020-08-30

**Authors:** Unai Galicia-Garcia, Asier Benito-Vicente, Shifa Jebari, Asier Larrea-Sebal, Haziq Siddiqi, Kepa B. Uribe, Helena Ostolaza, César Martín

**Affiliations:** 1 Fundación Biofisika Bizkaia, Barrio Sarriena s/n., 48940 Leioa (Bizkaia), Spain; u.galiciag@gmail.com (U.G.-G.); asierlarrea@yahoo.es (A.L.-S.); 2 Biofisika Institute (UPV/EHU, CSIC), Barrio Sarriena s/n., 48940 Leioa (Bizkaia), Spain; asier.benito@ehu.eus (A.B.-V.); sjebari001@ikasle.ehu.eus (S.J.); ofbmaplc@ehu.es (H.O.); 3 Department of Biochemistry and Molecular Biology, Universidad del País Vasco UPV/EHU, Apdo. 644, 48080 Bilbao, Spain; 4 Havard Medical School, 25 Shattuck St, Boston, MA 02115, USA; siddiqi.haziq1@gmail.com; 5 Center for Cooperative Research in Biomaterials (CIC biomaGUNE), Basque Research and Technology Alliance (BRTA), Paseo de Miramon 182, 20014 Donostia San Sebastián, Spain; kbelloso@cicbiomagune.es

**Keywords:** type 2 diabetes mellitus, insulin resistance, β-cell, liver, adipocyte, muscle, cardiovascular disease, pathophysiology

## Abstract

Type 2 Diabetes Mellitus (T2DM), one of the most common metabolic disorders, is caused by a combination of two primary factors: defective insulin secretion by pancreatic β-cells and the inability of insulin-sensitive tissues to respond appropriately to insulin. Because insulin release and activity are essential processes for glucose homeostasis, the molecular mechanisms involved in the synthesis and release of insulin, as well as in its detection are tightly regulated. Defects in any of the mechanisms involved in these processes can lead to a metabolic imbalance responsible for the development of the disease. This review analyzes the key aspects of T2DM, as well as the molecular mechanisms and pathways implicated in insulin metabolism leading to T2DM and insulin resistance. For that purpose, we summarize the data gathered up until now, focusing especially on insulin synthesis, insulin release, insulin sensing and on the downstream effects on individual insulin-sensitive organs. The review also covers the pathological conditions perpetuating T2DM such as nutritional factors, physical activity, gut dysbiosis and metabolic memory. Additionally, because T2DM is associated with accelerated atherosclerosis development, we review here some of the molecular mechanisms that link T2DM and insulin resistance (IR) as well as cardiovascular risk as one of the most important complications in T2DM.

## 1. Introduction

Type 2 Diabetes Mellitus (T2DM) is one of the most common metabolic disorders worldwide and its development is primarily caused by a combination of two main factors: defective insulin secretion by pancreatic β-cells and the inability of insulin-sensitive tissues to respond to insulin [1]. Insulin release and action have to precisely meet the metabolic demand; hence, the molecular mechanisms involved in the synthesis and release of insulin, as well as the insulin response in tissues must be tightly regulated. Therefore, defects in any of the mechanisms involved can lead to a metabolic imbalance that leads to the pathogenesis of T2DM.

This review analyses the key aspects of T2DM, as well as the molecular mechanisms and pathways implicated in insulin metabolism and associations between T2DM and cardiovascular pathophysiology. In this review, we describe the global trends of T2DM, the roles of major risk factors, in particular, obesity, lifestyle factors, genetic predispositions, gut dysbiosis, epigenetics and mitochondrial deregulation. We highlight physiological and molecular mechanisms leading to T2DM and its complications.

## 2. Type 2 Diabetes Mellitus: Background and Epidemiology

According to the World Health Organization (WHO) diabetes mellitus is a chronic, metabolic disease characterized by elevated levels of blood glucose, which leads over time to damage to the heart, vasculature, eyes, kidneys and nerves. Over 90% of diabetes mellitus cases are T2DM, a condition marked by deficient insulin secretion by pancreatic islet β-cells, tissue insulin resistance (IR) and an inadequate compensatory insulin secretory response [2,3]. Progression of the disease makes insulin secretion unable to maintain glucose homeostasis, producing hyperglycaemia. Patients with T2DM are mostly characterized by being obese or having a higher body fat percentage, distributed predominantly in the abdominal region. In this condition, adipose tissue promotes IR through various inflammatory mechanisms, including increased free fatty acid (FFA) release and adipokine deregulation. The main drivers of the T2DM epidemic are the global rise in obesity, sedentary lifestyles, high caloric diets and population aging, which have quadrupled the incidence and prevalence of T2DM [4,5].

The organs involved in T2DM development include the pancreas (β-cells and α-cells), liver, skeletal muscle, kidneys, brain, small intestine, and adipose tissue [6]. Evolving data suggest a role for adipokine dysregulation, inflammation, and abnormalities in gut microbiota, immune dysregulation, and inflammation have emerged as important pathophysiological factors [7].

Epidemiological data show alarming values that predict a worrisome projected future for T2DM. According to the International Diabetes Federation (IDF), in 2019, diabetes caused 4.2 million deaths; and 463 million adults aged between 20 and 79 years old were living with diabetes, a number that will likely rise up to 700 million by 2045. Diabetes was the underlying cause of at least 720 billion USD in health expenditure in 2019. Additionally, the true disease burden of T2DM is likely an underrepresentation as 1 in 3 diabetic people were underdiagnosed, equivalent to 232 million people. The greatest number of people suffering from diabetes are aged between 40 and 59 years old. Incidence and prevalence of T2DM vary according to geographical region, with more than 80% of patients living in low-to-middle-income countries, which poses additional challenges in effective treatment. Patients with T2DM have a 15% increased risk of all-cause mortality compared with people without diabetes with cardiovascular disease (CVD) as the greatest cause of morbidity and mortality associated with T2DM [8]. The association of diabetes with increased risk of coronary heart disease (hazard ratio [HR] 2.00; 95% CI 1.83–2.19), ischaemic stroke (HR 2.27; 1.95–2.65), and other vascular disease-related deaths (HR 1.73; 1.51–1.98) has been shown in a meta-analysis [9].

Epidemiology of T2DM is affected both by genetics and the environment. Genetic factors exert their effect following exposure to an environment characterized by sedentary behavior and high-calorie intake. Common glycaemic genetic variants for T2DM have been identified by genome-wide association studies, but these only account for 10% of total trait variance, suggesting that rare variants are important [10]. People of different ethnic origins may have different specific phenotypes that increase predisposition to clusters of CVD risk factors, including hypertension, insulin resistance, and dyslipidemia [11].

## 3. Risk Factors and Pathophysiology

T2DM risk factors include a complex combination of genetic, metabolic and environmental factors that interact with one another contributing to its prevalence. Although individual predisposition to T2DM due to non-modifiable risk factors (ethnicity and family history/genetic predisposition) has a strong genetic basis, evidence from epidemiological studies suggests that many cases of T2DM can be prevented by improving the main modifiable risk factors (obesity, low physical activity and an unhealthy diet) [12,13].

### 3.1. Ethnicity and Family History/Genetic Predisposition

Globally, the incidence and prevalence of T2DM are found to vary widely depending on ethnicity and geographical region with Japanese, Hispanics and Native Americans having the highest risks [14,15,16]. It has been shown higher incidence rates in Asians compared with a White American population [17,18], and white population in the UK, [19], where the highest risk is among the black population [20]. Whilst no clear reasons have been found, contributing factors such as modern lifestyle factors (which promote obesity), socioeconomic and direct genetic propensity or gene environmental interactions have been postulated.

Genetic predisposition plays an important part in the risk of developing T2DM. Over the past decade, several T2DM genome-wide association studies have shown the complex polygenic nature of T2DM in which most of these loci increase T2DM risk through primary effects on insulin secretion, and a minority act through reducing insulin action [21,22]. Dimas et al. grouped these variants on the basis of their potential intermediate mechanisms in T2DM pathophysiology, with four variants fitting a clear IR pattern; two reducing insulin secretion with fasting hyperglycemia; nine lowering insulin secretion with normal fasting glycemia; and one altering insulin processing [23]. According to these data, the genetic architecture of T2DM is highly polygenic, and additional association studies are needed to identify most T2DM loci [24]. Interactions between susceptibility loci and environmental factors could underlie the missing heritability of T2DM thus the impact of a given genetic variant can be modulated by the environmental factors (and vice versa) as evidenced by both observational studies and clinical trials [25].

### 3.2. Obesity, Low Physical Activity and Unhealthy Diet

Obesity (body-mass index [BMI]≥30 kg/m^2^) is the strongest risk factor for T2DM [26,27] and is associated with metabolic abnormalities resulting in IR [28]. There exist an inverse linear relationship between BMI and the age at diagnosis of T2DM [29]. The exact mechanisms by which obesity induces T2DM and IR remain to be elucidated; however, numerous factors have shown a significant role in the development of this pathological process, which involves both cell-autonomous mechanisms and inter-organ communications.

A sedentary lifestyle is another risk factor for T2DM as shown by the Women’s Health Study and in the Kuipio Ischemic Heart Disease Risk Factor Study, which showed a reduction of 34% and 56% reduction of developing T2DM in participants walking 2–3 h a week or at least 40 min a week, respectively [30,31]. There are three primary benefits of physical activity on the delay of T2DM onset. First, the contraction of skeletal muscle cells induces an increase in blood flow into the muscle, enhancing glucose uptake from plasma [32]. Second, physical activity reduces the notorious intra-abdominal fat, which is a known risk factor that promotes IR [33]. Finally, moderate-intensity exercise has been shown to improve glucose uptake by 40% [34]. Physical activity improves glucose uptake and insulin sensitivity but it can also improve or even reverse inflammation and oxidative stress, which are T2DM predisposing factors [32].

### 3.3. Pathophysiology

Regarding the pathophysiology of the disease, a malfunctioning of the feedback loops between insulin action and insulin secretion results in abnormally high glucose levels in blood [2]. In the case of β-cell dysfunction, insulin secretion is reduced, limiting the body’s capacity to maintain physiological glucose levels. On the other hand, IR contributes to increased glucose production in the liver and decreased glucose uptake both in the muscle, liver and adipose tissue. Even if both processes take place early in the pathogenesis and contribute to the development of the disease, β-cell dysfunction is usually more severe than IR. However, when both β-cell dysfunction and IR are present, hyperglycaemia is amplified leading to the progression of T2DM [35,36].

## 4. Mechanisms Leading to T2DM and Pathophysiology

### 4.1. Insulin Secretion: Physiological and Dysfunctional Mechanisms Leading to T2DM

#### 4.1.1. β-Cell Physiology

To safeguard proper β-cell function, cellular integrity must be ensured and the mechanisms and pathways implicated in the physiology of β-cell must be tightly regulated [35].

β-cells are responsible for insulin production, which is synthesized as pre-proinsulin. In the maturation process, pre-proinsulin undergoes a conformational modification carried out with the help of several proteins in the endoplasmic reticulum (ER) to yield proinsulin [37]. Afterwards, proinsulin is translocated from the ER to the Golgi apparatus (GA), entering into immature secretory vesicles and being cleaved into C-peptide and insulin [38,39].

**Figure 1:**
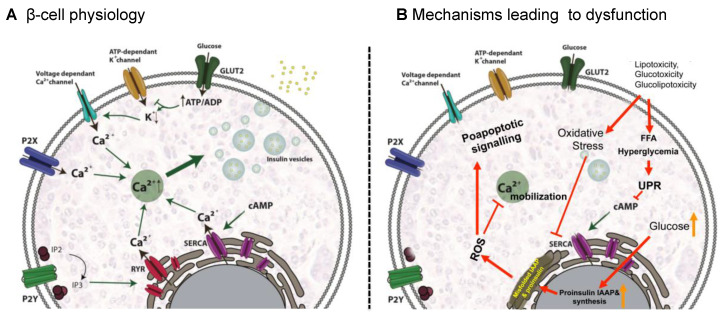
Signaling pathways involved in insulin secretion in β-cells in physiological conditions (**A**) and mechanisms leading to dysfunction (**B**). (**A**) Insulin release is primarily triggered by a response to high glucose concentrations and glucose in mainly internalized mainly through GLUT2 transporter. Glucose catabolism increases ATP/ADP ratio, ATP-dependant potassium channels are closed leading to membrane depolarization and opening of the voltage dependant Ca^2+^ channels. The latter enables Ca^2+^ influx triggering insulin exocytosis. Additional Ca^2+^ channels as P2X, P2Y, SERCA and RYR contribute to Ca^2+^ mobilization and insulin secretion. (**B**) hyperglycemia and hyperlipidemia promote oxidative stress leading to ROS generation that inhibits Ca^2+^ mobilization and activates proapoptotic signals. Additionally, an excess of FFAs and hyperglycemia lead to the activation of the apoptotic unfolded protein response (UPR) pathways and generation of ER stress. Sustained high glucose levels increase proinsulin and IAAP biosynthesis, which generate ROS. GLUT2: glucose transporter 2, P2X: purinergic receptor X; P2Y: purinergic receptor Y; IP2: inositol 1,3-bisphosphate; IP3: inositol 1,4,5-trisphosphate; RYR: ryanodine receptor channel; SERCA: sarco-endoplasmic reticulum Ca^2+^-ATPase; FFA: free fatty acid, ROS: reactive oxygen species; UPR: unfolded protein response.

Once matured, insulin is stored in granules until insulin release is triggered. Insulin release is primarily triggered by a response to high glucose concentrations. It is worth noting that some other factors can also induce insulin release such as amino acids, fatty acids and hormones [40]. When circulating glucose levels increase, β-cells take in glucose mainly through the glucose transporter 2 (GLUT2), a solute carrier protein that also works as a glucose sensor for β-cells. Once glucose enters, glucose catabolism is activated, increasing the intracellular ATP/ADP ratio, which induces the closing of ATP-dependant potassium channels in the plasma membrane. This leads to membrane depolarization and opening of the voltage dependant Ca^2+^ channels, enabling Ca^2+^ to enter the cell. The rise in the intracellular Ca^2+^ concentration triggers the priming and fusion of the secretory insulin-containing granules to the plasma membrane, resulting in insulin exocytosis [38,40,41,42] (Figure 1A).

Additionally, Ca^2+^ signals can be amplified by the RY receptors (RYR) and may play important roles in stimulus- insulin secretion coupling by virtue of their strategic locations within the cell and their ability to mediate Ca^2+^ induced Ca^2+^ release (CICR). RYR amplifies Ca^2+^ signals when the channel is sensitized by messenger molecules generated from the nutrient metabolism or ligand-binding and are involved in the amplification of insulin secretion [43] (Figure 1A).

Nevertheless, other cell signals can also assist or enhance insulin release from β-cells. Among them, cAMP might be the most important messenger potentiating insulin release. Accumulated evidence suggests that cAMP induces insulin-containing secretory vesicle mobilization by depleting intracellular Ca^2+^ reservoirs, thereby increasing intracellular Ca^2+^ concentrations [44]. There is also compelling evidence that extracellular ATP is another important regulator of β-cell function. It is well-documented that β-cells release ATP through exocytosis of insulin granules upon glucose stimulation. Purinergic signaling via P2Y and P2X purinergic receptors stimulates Ca^2+^ mobilization and regulates insulin exocytosis also independently of glucose. P2Y purinoreceptors have been reported to be coupled to G-proteins [45,46] whereas P2X-type receptors are ATP-activated ligand-gated ion channels non-selective for cations [47]. In the case of P2Y receptors, it has been proposed that insulin release could be mediated by intracellular Ca^2+^ mobilization in response to inositol-1,4,5-trisphosphate (IP3) formation that triggers the release of Ca^2+^ from ER stores, which amplifies the exocytosis-triggering Ca^2+^ signal [48,49] (Figure 1A).

#### 4.1.2. Mechanisms Leading to β-Cell Dysfunction

β-cell dysfunction has been traditionally associated with β-cell death [50]. However, recent evidence suggests that the dysfunction of β-cells in T2DM might be due to a more complex network of interactions between the environment and different molecular pathways implicated in cell biology [51]. In an excessive nutritional state, similar to that found in obesity, hyperglycemia and hyperlipidemia are often present, favoring IR and chronic inflammation. Under these circumstances, β-cells, due to differences in their genetic susceptibility, are subject to toxic pressures including inflammation, inflammatory stress, ER stress, metabolic/oxidative stress, amyloid stress, with the potential of ultimately leading to a loss of islet integrity [50].

An excess of FFAs and hyperglycemia lead to β-cell dysfunction by inducing ER stress through the activation of the apoptotic unfolded protein response (UPR) pathways [52]. In fact, lipotoxicity, glucotoxicity and glucolipotoxicity occurring in obesity, induce metabolic and oxidative stress that leads to β-cell damage [51]. Stress derived from high levels of saturated FFAs can activate the UPR pathway by several mechanisms including inhibition of the sarco/endoplasmic reticulum Ca^2+^ ATPase (SERCA) responsible for ER Ca^2+^ mobilization; activation of IP3 receptors or direct impairment of ER homeostasis. In addition, sustained high glucose levels increase proinsulin biosynthesis and islet amyloid polypeptides (IAAP) in β-cells, leading to the accumulation of misfolded insulin and IAAP and increasing the production of oxidative protein folding-mediated reactive oxygen species (ROS) [52]. These effects alter physiological ER Ca^2+^ mobilization and favor proapoptotic signals, proinsulin mRNA degradation and induce interleukin (IL)-1 β release that recruits macrophages and enhances local islet inflammation [51] (Figure 1B).

As previously mentioned, insulin secretion has to be finely regulated to precisely meet metabolic demand. For that reason, proper islet integrity must be conserved in order to allow β-cells to respond to metabolic needs. Under pathogenic conditions, the mechanism described above can ultimately lead to disruption of islet integrity/organization, impairing optimal cell-to-cell communication within pancreatic islets, contributing to poor regulation of insulin and glucagon release and ultimately exacerbating the hyperglycemia. Defects in the synthesis of any insulin precursors, or insulin itself, as well as disruption of the secretion mechanism, can lead to insulin secretory dysfunction, the primary driver of β-cell failure, and a foundation of T2DM. For instance, reduced expression in the GLUT2 glucose transporter would affect the downstream signaling pathway [53], while failure in the folding of proinsulin is another finding commonly linked to deficient insulin production and diabetes [54].

#### 4.1.3. Pathological Conditions Perpetuating T2DM

##### Nutritional Factors

High-caloric Western diet contains large amounts of fats and carbohydrates that elevate blood glucose and circulating very-low-density lipoproteins (VLDLs), chylomicrons (CMs) and their remnants (CMRs) that are rich in triglycerides (TG). This induces a spike in reactive oxygen species (ROS) concentrations, which in turn leads to an abnormal generation of inflammatory molecules. Given that inflammation is a recognized inducer of oxidative stress, a synergistic interaction occurs between the two processes after a heavy meal, with consequent amplification of harmful postprandial effects. The sustained and marked increase in steady-state levels of ROS contributes significantly to the pathogenesis of T2DM and IR. Therefore, a pro-oxidant environment leads to mitochondrial dysfunction, ER stress, activation of NADPH oxidase (NOX) and superoxide (O_2_^−^) production. The increase in O_2_^−^ production activates the five major pathways involved in the pathogenesis of diabetes complications: enhancement of the polyol pathway, increased formation of advanced glycation end products (AGEs), increased expression of AGEs receptor and its activating ligands, activation of protein kinase C (PKC) isoforms, and overactivity of the hexosamine pathway [55,56,57]. Through these pathways, increased intracellular ROS causes defective angiogenesis in response to ischemia, activates a number of proinflammatory pathways, and cause long-lasting epigenetic changes which drive persistent expression of proinflammatory genes even after glycemia is normalized [58]. Additionally, increased blood levels of FFAs also lead to mitochondrial dysfunction through two different mechanisms: (1) FFA metabolism by-products disturb the electron flow throughout the mitochondrial respiratory chain and (2) through the incorporation of FFAs into the mitochondrial membranes, thus likely favoring electron leakage [59].

##### Physical Activity

Reduced physical activity and exercise training, and increased sedentary behaviors constitute a link between obesity and T2DM and are associated with increased markers of chronic low-grade systemic inflammation [60,61]. In this condition, proinflammatory molecules are released into the bloodstream and within specific tissues such as interleukin 6 (IL-6), C-Reactive Protein (CRP), tumor necrosis factor-alpha (TNF-α) or IL-1 induces an inflammatory state known as metabolic inflammation [37]. Indeed, IL-1 is involved in the autoimmune response to β-cells in the pancreas, inhibition of β-cell function and activation of the nuclear factor kappa-light-chain-enhancer of activated B cells (NF-κB) transcription factor, thus inhibiting β-cell function and promoting apoptosis [32]. Preclinical data suggest that inflammation resolution could prevent the development of T2DM in obesity and prediabetes, which was substantiated by preclinical animal data showing that deletion of the macromolecular complex NLRP3 inflammasome, responsible for the production of IL-1β and IL-18, resulted in improved insulin sensitivity [62].

Intentional weight loss remains the cornerstone therapy to improve insulin sensitivity and in some circumstances to prevent the incidence of T2DM in individuals with obesity and prediabetes [63]. Regular exercise and increased physical activity enhance the production of anti-inflammatory cytokines such as IL-1 Receptor antagonist (IL-1Ra) and soluble TNF receptor (s-TNF-R) that are antagonists of IL-1 and TNF-α, respectively. Individuals with increased physical activity also show reduced circulating levels of IL-6, IL-18 and CRP, together with lower levels of leptin, a molecule associated with CRP [64]. Physical exercise can improve T2DM-inducing oxidative stress by inducing the synthesis of antioxidants such as glutathione (GSH), a major non-enzymatic antioxidant [65] and other antioxidant enzymes which lead to a long-term reduction in free radical levels [32].

Finally, irisin is an exercise-regulated myokine, which improves glucose tolerance [66] secreted by skeletal muscle [67] and adipose tissue [57] in response to exercise [68]. T2DM patients have been found to have lower circulating levels of irisin compared to control subjects. Additionally, diabetic patients with CVD had significantly lower serum irisin than non-CVD patients [69]. Low levels of serum irisin have been associated with 1.6 times increased risk of CVD incidence in T2DM patients [70].

##### Gut Dysbiosis

Gut microbiota is composed of many microbial species that impact human physiology and participate in different biological processes [71]. They can modulate the immune system and inflammatory response, regulate gut barrier integrity and human metabolism, take part in the synthesis of metabolites. Gut resident microorganisms produce many metabolites that contribute to physiology in healthy individuals. However, changes due to both inherited and acquired factors such as age, nutrition, lifestyle, genetic predisposition, or underlying diseases can affect the gut microbiota produced metabolite proportion leading to metabolic disturbances that can culminate in disease [72,73,74]. The better understating of gut microbiota has evidenced its important role in the development of diabetes and recent studies indicate that changes in dysbiosis can promote IR and T2DM [75]. A high-fat diet can induce up to threefold lipopolysaccharide (from Gram-negative bacteria) production in mice models, thereby contributing to low-grade inflammation and insulin resistance [76,77]. Furthermore, intestinal dysbiosis can reduce short-chain fatty acid synthesis that promotes gut barrier integrity, pancreatic β-cell proliferation and insulin biosynthesis [78,79]. Dysbiosis can also compromise the production of other metabolites such as branched aminoacids and trimethylamine thus disrupting glucose homeostasis and triggering T2DM development [80,81]. Understanding the clinical implications of the gut microbiome is a relatively new field, and requires further research to better elucidate the connection between gut microbiota and T2DM.

##### Metabolic Memory

Metabolic memory refers to the persistence of diabetic complications even after maintained glycemic control. This concept arose from the results of multiple large-scale clinical trials, which showed that after diabetes onset, diabetes complications persist and progress even when glycemic control is restored through pharmaceutical intervention [82,83,84]. Among them, the UKPDS post-trial study and Steno-2 trial showed that specifically early glycemic interventions prevent diabetic complication and has a marked decrease in CVD endpoints in patients that received either standard or intensive treatment following their diagnosis [84]. Later on, animal models of diabetes and in vitro cell cultures demonstrated that the initial hyperglycemic period results in permanent abnormalities (including aberrant gene expression) of target organs/cells [85,86,87,88]. Metabolic memory involves four mechanisms: epigenetics, oxidative stress, non-enzymatic glycation of proteins and chronic inflammation.

Epigenetics involves genetic modulation by factors other than individuals’ DNA sequence, and can regulate gene expression and determine which proteins are transcribed [89]. There are different epigenetic regulation mechanisms: direct methylation of cytosine or adenine residues, covalent modifications of histone proteins, higher-order chromatin structure and non-coding RNAs. Disruptions or imbalances in epigenetic mechanisms can lead to the development of diabetic pathophysiology [90].

MicroRNAs (miRNAs) are small non-coding RNA sequences synthesized as non-mature molecules that undergo several processing steps both in the nucleus and in the cytoplasm to become fully matured miRNAs. Once matured, miRNAs bind to their target gene’s mRNA, leading to mRNA silencing or degradation [91]. Increasing evidence highlights the importance of miRNA mediated post-transcriptional regulation in different aspects of β-cell biology such as cell differentiation, cytokine and growth factor-mediated signaling, glucose metabolism and insulin synthesis and secretion [92]. Deregulation of miRNA expression can directly impair β-cell function leading to the development of T2DM [93]. To date, more than 2600 miRNAs have been described within the human genome (miRBase, v.22.1), and multiple miRNAs have been shown to be involved in the pathogenesis of T2DM, including miR-200, miR-7, miR-184, miR-212/miR132 and miR-130a/b/miR-152 [94]. For instance, overexpression of miR-7 results in reduced insulin secretion via inhibition of genes involved in vesicle fusion and SNARE activity such as Snca, Cspa and Cplx1 [95]. In the case of miR-375, over-expression results in impaired exocytosis and thereby reduced insulin secretion. Conversely, it is the downregulation of miR-375 expression that causes a reduction in β-cell mass [93,96,97].

Several studies have evidenced that deregulation of the microRNA (miRNA) profile (post-translational histone methylation and non-canonical histone variant inclusion in octomers) may persist even after normoglycemia restoration [98,99,100,101]. MiRNAs participate in metabolic memory by targeting the mRNA of genes encoding enzymes involved in DNA methylation and those tightly regulated at the level of promoter methylation, transcription, and processing [102]. It has been shown that high glucose levels can alter post-translational histone modifications (PTHMs) and the activity of DNA methyltransferases generating irreversible changes that explain the long-term harmful effects of metabolic memory [103,104,105,106]. 

Hyperglycaemia induces an excess of ROS generation by mitochondria, which gives rise to diabetes complications [107] that may persist even when hyperglycemia is controlled. The damage following hyperglycemia-induced oxidative stress can be prevented when good glycemic control is initiated very early, but is not easily reversed if poor control is maintained for a longer duration [108,109]. At the early stages of T2DM, there is a relationship between hyperglycemia, increased oxidative stress, and excessive AGE formation. As the disease progresses, there is persistent protein glycation of the components of the respiratory chain that together with mitochondrial DNA damage can generate a hyperglycemia-independent concatenation of events leading to a synergy between oxidative stress and AGEs [86]. The effects of this metabolic imbalance activate inflammatory processes through receptor binding of AGEs or ROS which can modify the composition and structure of the extracellular matrix [98]. These structural changes may cause endothelial dysfunction and then atherosclerosis [98].

Finally, low-grade inflammation, which is involved in T2DM development and its vascular complications, has been shown to mediate metabolic memory. Many environmental factors (age, obesity, sedentarism and diet) that promote T2DM development trigger an inflammatory response leading to IR and endothelial dysfunction [105,110,111]. Obesity leads to NF-κB activation, which mediates the expression of inflammatory genes, which enhances monocyte binding to endothelial and vascular smooth muscle cells, subsequently promoting monocyte-to-macrophage differentiation [105]. In addition, NF-κB activation induces expression of inflammatory cytokines that are involved in vascular inflammation, with subsequent generation of endothelial adhesion molecules, proteases, and other mediators [111]. Another important factor that links inflammation and oxidative stress in obesity condition is the Toll-like receptor, which contributes to hypertension, insulin resistance, and obesity [105].

**Figure 2:**
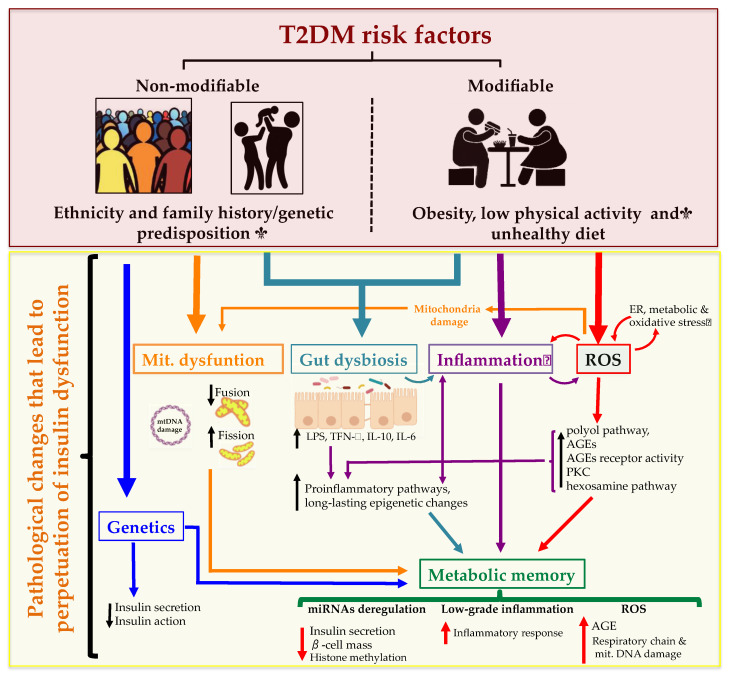
Type 2 Diabetes Mellitus (T2DM) risk factors and the pathological changes leading to the perpetuation of insulin dysfunction. Complex combinations of genetic, metabolic and environmental factors that interact with one another constitute both non-modifiable (ethnicity and family history/genetic predisposition) and modifiable risk factors (obesity, low physical activity and an unhealthy diet). These states affect cell function resulting in a complex network of pathological changes that influence mutually and lead to the perpetuation of insulin dysfunction. ROS: reactive oxygen species; ER: endoplasmic reticulum; AGEs: advanced glycation end products; PKC: protein kinase C; LPS: lipopolysaccharide; miRNA: microRNA.

In summary, T2DM is a heterogeneous and progressive disorder that represents a series of metabolic conditions associated with hyperglycemia and caused by defects in insulin secretion and/or insulin action due to a complex network of pathological conditions. There are many different paths, driven by various genetic and environmental factors, that interact and mutually reinforce each other leading to an increased risk of other diseases including heart, peripheral arterial and cerebrovascular disease, obesity and nonalcoholic fatty liver disease, among others. The complex network of pathological conditions leading to T2DM development are summarized in Figure 2.

##### Mitochondrial Dysfunction

**Figure 3:**
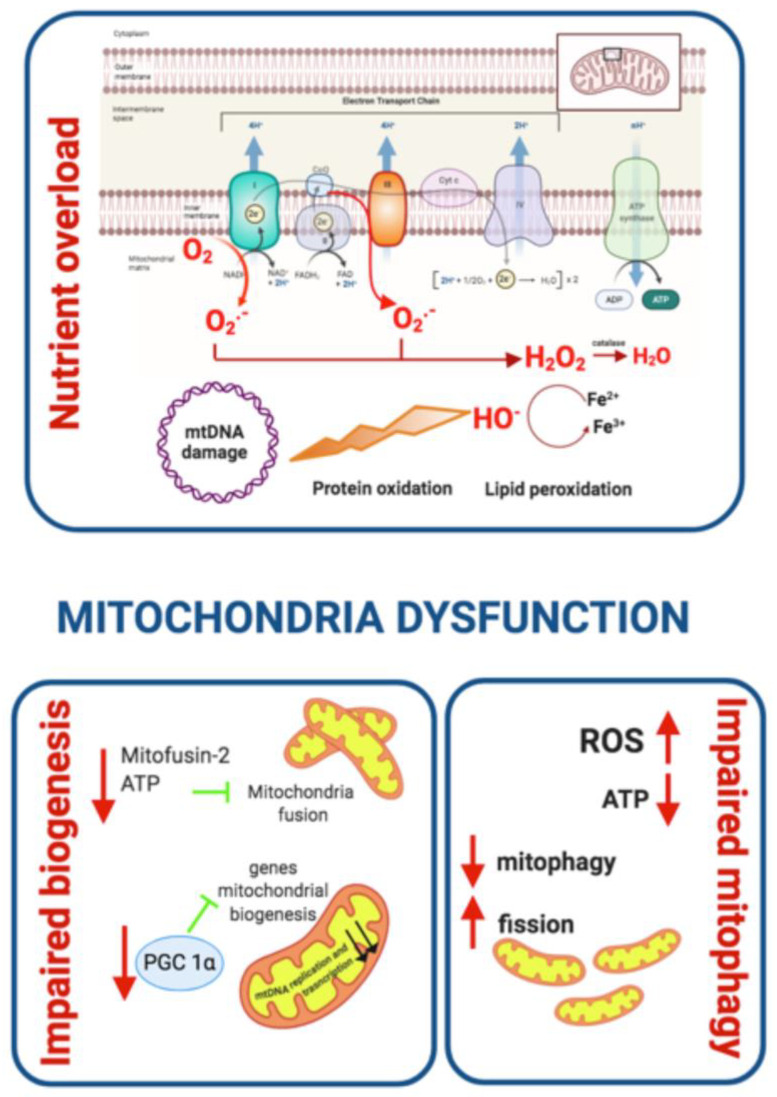
Mitochondrial dysfunction and contribution to T2DM development. Oxidative stress, defective mitochondrial biogenesis and impaired mitophagy promote mitochondrial dysfunction. Generation of ROS links mitochondrial dysfunction and IR. As a consequence of nutrient overload, electron supply to the mitochondrial ETC increases and the electron excess is transferred to oxygen generating O_2_^−^ and H_2_O_2_. ROS oxidize proteins, damage DNA and membrane lipids. Mitofusin-2 and PGC 1α are downregulated leading to reduced mitochondrial biogenesis. Cellular stress and ROS production contribute to higher mitochondrial fission and impaired mitophagy. PCG 1α: Peroxisome proliferator-activated receptor-gamma coactivator-1.

There is increasing evidence associating mitochondrial dysfunction with T2DM development, age-related IR and T2DM complications [112]. Indeed, oxidative stress, defective mitochondrial biogenesis, genetic mutations affecting mitochondrial integrity and aging promote mitochondrial dysfunction and are closely associated with T2DM development (Figure 3) [113,114].

The main function of mitochondria is ATP synthesis through oxidative phosphorylation in response to metabolic demand [115]. Mitochondria also participate in the production of different metabolites used as precursors of several macromolecules (lipids, proteins, and DNA). In addition, mitochondria play an important role in maintaining ion homeostasis, ROS clearance, the stress response, and serve to integrate multiple signaling pathways [116,117]. An imbalance between energy intake and expenditure in the mitochondria generates mitochondrial dysfunction, a state characterized by a reduced ratio of energy production to respiration [112]. Under these circumstances, nutrient oxidation efficiency is reduced leading to a decreased ratio of ATP synthesis/oxygen consumption, which increases O_2_^−^ production [118]. In fact, the accumulation of ROS in the mitochondria is one proposed mechanism linking mitochondrial dysfunction to IR [119]. This relationship was corroborated in studies showing decreased mitochondria oxidative capacity in skeletal muscle and impaired lipid metabolism in obese and insulin-resistant individuals compared to healthy controls [120,121,122]. In addition, patients with T2DM have been found to have downregulation of genes involved in oxidative metabolism that are regulated by the peroxisome proliferator-activated receptor γ co-activator 1α (PGC 1α) [123,124] and a diminished phosphocreatine re-synthesis rate, both indicative of impaired mitochondrial function [125] (Figure 3). Moreover, some relatives of T2DM patients have been found to have decreased mitochondrial respiration suggesting that mitochondrial dysfunction may precede T2DM development. It has also been proposed that T2DM development may be a direct consequence of defects in the oxidative phosphorylation system and the electron transport chain (ETC) rather than a decrease in mitochondrial content [126].

The generation of ROS is highly implicated in the relationship between mitochondrial dysfunction and insulin resistance. ROS production takes place mainly at complex I and complex III of the ETC and increases when ETC is not able to handle excessive electron input. In these circumstances, as a consequence of nutrient overload, electron supply to the mitochondrial ETC increases and the electron excess is transferred to oxygen generating O_2_^−^ and subsequent hydrogen peroxide [127]. ROS generated in mitochondria oxidize the Cys and Met residues in proteins, damaging protein structure, impairing their function and eventually causing cell death. ROS species also damage DNA and membrane lipids, thus promoting mitochondrial dysfunction [128]. In addition, ROS overproduction activates the polyol pathway, the formation of AGEs, and the expression of the AGEs receptor and its activating ligands. It also activates PKC isoforms and upregulates the hexosamine pathway contributing to T2DM worsening [129,130]. In sum, excessive ROS generation by mitochondria contributes to accelerated T2DM progression (Figure 3).

Mitochondrial dysfunction includes a reduction in mitochondrial biogenesis, along with a decrease in the expression of mitochondrial oxidative proteins, such as ETC complexes, which leads to decreased substrate oxidation. The damage produced by high oxidative stress in the mitochondria activates mitophagic processes in order to eliminate dysfunctional mitochondria or in case of excessive cellular stress to apoptosis [131]. These two processes reduce substrate utilization and enhance the accumulation of lipid intermediates such as diacylglycerols (DAG) and ceramide (CER) that disrupt the insulin signaling pathway [132]. DAG induces an increment of the serine/threonine phosphorylation of IRS-1, reducing its insulin-stimulated tyrosine phosphorylation and downstream propagation of the insulin signaling pathway [133] while CER inhibits protein kinase AKT [133]. The accumulation of DAG and CER contributes to the mitochondrial dysfunction seen in IR (Figure 3).

Defects in mitochondrial biogenesis may be mediated by the downregulation of PGC 1α that has also been detected in T2DM patients [123,124]. PGC 1α is a transcription coactivator that regulates the expression of key genes involved in mitochondrial biogenesis, adaptive thermogenesis and metabolic substrate metabolism [134]. Furthermore, some of the genes involved in oxidative metabolism that are downregulated in individuals with T2DM are under the control of PGC 1α [124]. *Mitofusin-2*, a key driver in mitochondria biogenesis is also downregulated in humans with T2DM [135]. Interestingly, *mitofusin-2* levels increase upon weight loss indicating that nutrient and energy oversupply leads to mitochondrial dynamics defects [135].

Mitochondrial homeostasis is maintained via mitochondrial biogenesis and the selective clearance of damaged organelles. Mitochondrial dynamics are crucial to maintaining healthy mitochondria and control their quantity. Mitochondria fission promotes the removal of damaged mitochondria in a process known as mitophagy, which has to be efficiently and tightly regulated in order to preserve cell homeostasis [136]. Thus, mitophagy is considered to be one of the core mechanisms controlling the mitochondrial quantity and quality [137]. The process of removing damaged mitochondria consists of two steps: the induction of general autophagy and the priming of damaged mitochondria for selective autophagy recognition [138]. Once the degradation process is completed, the products are released back into the cytosol where macromolecular constituents are recycled. This process generates energy to maintain cell viability under unfavorable conditions and protects the cell during stress conditions [136,139]. When mitophagy is impaired, cellular stress and ROS production increase contributing to reduced hepatic insulin sensitivity and glucose homeostasis, two of the major pathological branches of T2DM development [112,140]. Deregulation of mitochondria dynamics with a shift towards fission promotes metabolic dysfunction as demonstrated by the onset of obesity and IR following the ablation of fusion protein in mice [141,142]. Furthermore, increased mitochondrial fission and mitochondrial fragmentation have been associated with mitochondrial depolarization, impaired ATP production and decreased insulin-dependent glucose uptake as well as increased mitochondrial ROS and impaired insulin signalling in C2C12 murine cell line and cybrids, respectively [143,144]. These studies highlight that the deleterious effect of unbalanced mitochondrial dynamics on metabolic health. Enhanced mitochondria fission also negatively impacts fatty acid β-oxidation, which is a pivotal metabolic defect in obesity and IR [120,121] contributing to the accumulation of lipotoxic lipid species. Fusion-shifted mitochondria dynamics has been also associated with an increase in fatty acid utilization putatively preventing lipotoxicity [145].

The role of mitochondrial genetics in the risk of T2DM has been clearly established. Indeed, several mtDNA variants (homoplasmic or heteroplasmic) have been associated with T2DM development. To date, the group of heteroplasmic variants associated with a higher risk of T2DM development includes A3243G, T14577C and A5178C [146,147,148,149]. The group of homoplasmic variants associated with T2DM risk includes C1310T, G1438A, A12026G, T16189C and A14693G [150,151,152]. It is important to note that additional studies are necessary to determine whether more metabolically active tissues that generate more mitochondrial ROS have increased rates of mtDNA heteroplasmy in T2DM.

To summarize, there is a highly nuanced and bi-directional relationship between mitochondrial dysfunction and T2DM. On one hand, aspects of T2DM such as insulin resistance can lead to mitochondrial dysfunction, such as through nutrient overload leading to ROS accumulation. On the other hand, mitochondrial dysfunction may predispose patients to subsequently developing T2DM, as evidenced by the presence of mtDNA variants associated with T2DM. Additional research is needed to better characterize the relationship between mitochondrial health and diabetes.

## 5. Insulin Resistance

IR refers to a decrease in the metabolic response of insulin-responsive cells to insulin or, at a systemic level, an impaired/lower response to circulating insulin by blood glucose levels [153]. There are three broad categories of IR or insulin-deficient conditions: (1) diminished insulin secretion by β-cells; (2) insulin antagonists in the plasma, due either to counter-regulatory hormones or non-hormonal bodies that impair insulin receptors or signaling; and (3) impaired insulin response in target tissues [154]. The action of insulin is influenced by the interplay of additional molecules including growth hormone and IGF-1 in the fed state. While fasting, the insulin response is mitigated by glucagon, glucocorticoids and catecholamines in order to prevent insulin-induced hypoglycemia. The ratio of insulin/glucagon plays a major role in this regulation, since it determines the relative degree of phosphorylation of downstream enzymes in the regulatory signaling pathways. While catecholamines promote lipolysis and glycogenolysis, glucocorticoids promote muscle catabolism, gluconeogenesis and lipolysis. Hence, excessive secretion of these hormones may be responsible for inducing IR [155,156]. Regarding the last category, there are three main extra-pancreatic insulin-sensitive organs that play major roles in the aforementioned processes: skeletal muscle, adipose tissue and liver. A defective action of insulin in these tissues often precedes the development of systemic IR, thus progressively leading T2DM.

### 5.1. Skeletal Muscle

Skeletal muscle IR is considered to be the most important extra-pancreatic factor in the development of T2DM [157]. Under physiological conditions, insulin stimulates muscle glycogen synthesis by enhancing glucose uptake from plasma. There are three primary rate-limiting factors implicated in glucose uptake and glycogen synthesis: glycogen synthase, hexokinase and the glucose transporter GLUT4 [158]. Upon insulin binding to insulin receptor (INSR) in muscle cells, GLUT4 translocates from intracellular compartments (early endosomes (EE), endosomal recycling compartment (ERC) and trans-Golgi network (TGN)) to the plasma membrane. This process allows glucose uptake and reduces circulating glucose levels [159].

Mutations that reduce the expression of insulin receptor or GLUT4, as well as any defect in either upstream or downstream signaling pathway would reduce glucose intake into the muscle resulting in a hyperglycaemic state [153,160]. The activation of INSR tyrosine kinase activity is essential for the action of insulin on glucose metabolism. Insulin binding to the α-subunit of the INSR causes phosphorylation of the β-subunit on multiple tyrosine residues and allows insulin-mediated signaling. Thus, mutations in any of the main phosphorylation sites can impair INSR tyrosine kinase activity, thereby impairing insulin action on skeletal muscle [161]. As mentioned above, mutations in key proteins of the downstream signaling pathway such as IRS-1 and IRS-2 or phosphoinositide 3-kinase (PI3K) also impair insulin action on the muscle. Apart from mutations or defective epigenetic regulation, environmental factors can also play an important role in glucose uptake by muscle. Physical activity increases blood flow into skeletal muscle cells and thereby enhances glucose utilization [32]. Obesity, which is associated with chronic inflammation, contributes to IR and T2DM. Increasing evidence suggests that as a consequence of obesity, increased immune cell infiltration and secretion of proinflammatory molecules in intermyocellular and perimuscular adipose tissue leads to skeletal muscle inflammation. This ultimately leads to myocyte inflammation, impaired myocyte metabolism, and contributes to IR via paracrine effects [162].

### 5.2. Adipose Tissue

Adipose tissue is a metabolically dynamic tissue capable of synthesizing a wide range of biologically active compounds that regulate metabolic homeostasis at a systemic level [163]. Indeed, adipose tissue participates in a broad range of biological processes involving, among others, immunity, coagulation, angiogenesis, fibrinolysis, reproduction, vascular tone control, appetite regulation, body weight homeostasis and glucose and lipid metabolism [164].

Insulin acts on adipose tissue in two different ways: (1) stimulating glucose uptake and triglyceride synthesis; and (2) suppressing triglyceride hydrolysis and inducing the uptake of FFA and glycerol from circulation [165]. In the fed state, GLUT4 allows uptake of glucose from the bloodstream into adipocytes, activating glycolysis in which glycerol-3-phospate (glycerol-3-P) is produced and incorporated into lipogenic pathways. Glycerol-3-P, along with the fatty acids coming from VLDLs, are esterified, forming triacylglycerol (TGA) that is stored in lipid droplets. During metabolic stress, TGA droplets the adipocyte are depleted, in order to provide FFA to be used as an energy source in other tissues.

An impaired response to insulin stimulation by adipose tissue is known as adipose IR (Adipose-IR). Adipose-IR can lead to impaired suppression of lipolysis, impaired glucose uptake, and enhanced FFA release into plasma even in the presence of high insulin levels [166]. Among the signaling elements affected by adipose-IR, we found that defective AKT activation impairs GLUT4 translocation to the membrane and promotes the activation of lipolytic enzymes that aggravate hyperglycemia [153]. Adipose-IR, as mentioned before, is associated with glucose intolerance and elevated release of FFA into a plasma that accumulates in other tissues such as muscle or liver. In the case of the liver, FFA accumulation results in impaired insulin signaling that promotes hepatic gluconeogenesis and impairs the glucose-stimulated insulin response, inducing T2DM development.

**Figure 4:**
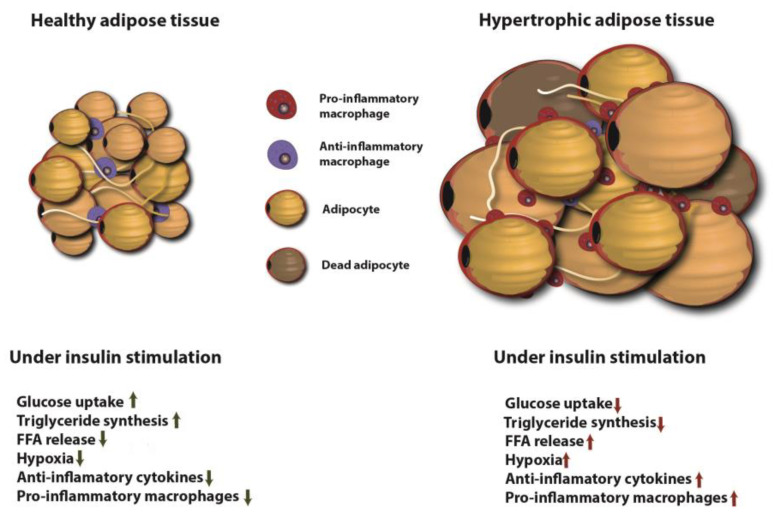
Insulin stimulation effects on healthy and hypertrophic adipose tissue. In healthy adipose tissue insulin stimulates glucose uptake and TG synthesis, induces FFA uptake and diminishes macrophage-mediated inflammation. Hypertrophic adipose tissue leads to diminished glucose uptake, TG synthesis and enhances FFA release, hypoxia and macrophage-mediated inflammation. FFA: free fatty acid.

It has been shown that abnormally increased adipose tissue mass and adipocyte size correlate with pathologic vascularisation, hypoxia, fibrosis and macrophage-mediated inflammation [167]. A high-fat diet and obesity can activate saturated FFA-stimulated adenine nucleotide translocase 2 (ANT2), an inner mitochondrial protein that results in adipocyte hypoxia and triggers the transcription factor hypoxia-inducible factor-1α (HIF-1α). This culminates in adipose tissue dysfunction and inflammation [1]. Hypertrophied adipocytes as well as adipose tissue-resident immune cells contribute to increased circulating levels of proinflammatory cytokines. This increase in circulating proinflammatory molecules, together with an increase in local cytokine releases such as TNF and IL-1β and IL-6 facilitates the emergence of a chronic state of low-grade systemic inflammation, also known as metabolic inflammation [1]. This chronic inflammatory state is considered to be a key part in the pathogenesis of IR and T2DM [168]. The insulin stimulation effects on healthy and hypertrophic adipose tissue are shown in Figure 4.

### 5.3. Liver

In the liver, insulin does not only regulate glucose production/utilization but also affects lipid metabolism more broadly. When circulating glucose levels increase and insulin is secreted by pancreatic β-cells, insulin binding to liver INSR induces autophosphorylation of the receptor. Consequently, insulin receptor substrates (IRSs) are recruited and phosphorylated. In turn, IRSs activate PI3K, which phosphorylates phosphatidylinositol (4,5)-bisphosphate (PIP2), generating phosphatidylinositol (3,4,5)-triphosphate (PIP3). PIP3 then activates PDK1, which phosphorylates AKT. In addition, AKT is phosphorylated by mTORC2. Once AKT is fully activated, it participates in several downstream pathways that regulate multiple metabolic processes including glycogen synthesis, gluconeogenesis, glycolysis and lipid synthesis [169].

In physiological states, the combined action of glucagon and insulin allows the precise regulation of hepatic glucose output. While glucagon induces hepatic glucose production, insulin acts as a potent inhibitor of glucose production when its concentration in the blood is elevated [170]. The effect of insulin on hepatic glucose production is due to both direct and indirect mechanisms. However, the relative importance of each of these mechanisms remains unclear [171].

**Figure 5:**
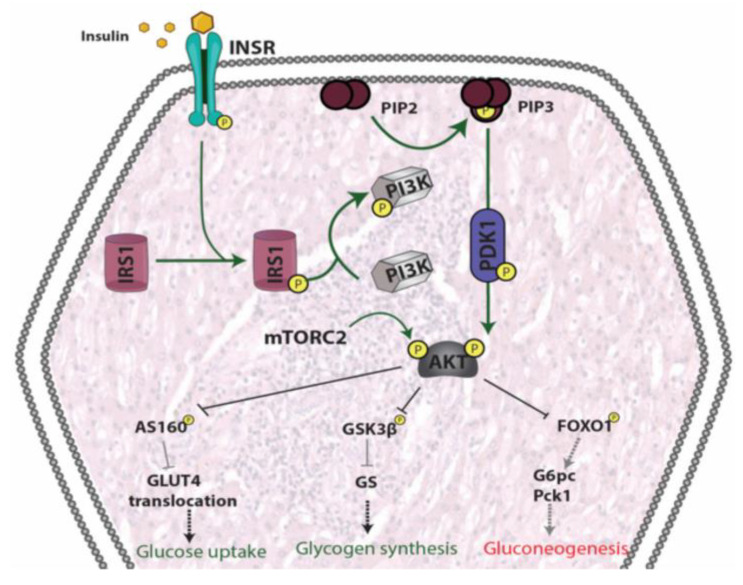
Signaling pathways involved in insulin signaling in hepatocytes. Binding of insulin to INSR induces IRSs recruitment and phosphorylation. Phosphorylated IRSs activate PI3K, generating PIP3 which activates PDK resulting in AKT phosphorylation. AKT is fully activated by further mTORC2 phosphorylation and participates in several downstream pathways that regulate multiple metabolic processes including glycogen synthesis, gluconeogenesis, glycolysis and lipid synthesis. INSR: insulin receptor; PIP2: inositol 1,3-bisphosphate; PIP3: inositol 1,4,5-trisphosphate; IRS1: insulin receptor substrate 1; PI3K: phosphoinositide 3 kinase; mTORC2: mammalian target of rapamycin complex 2; PDK1: Phosphoinositide-dependent kinase-1; AKT: protein kinase B; AS160: Akt substrate of 160 kDa; GLUT4: glucose transporter 4; GSk3β: Glycogen Synthase Kinase 3 Beta; GS: Glycogen synthase; FOXO1: Forkhead box protein O1; G6pc:Glucose 6 phosphate; Pck1: Phosphoenolpyruvate Carboxykinase 1.

In addition to inducing glycogen synthesis, insulin also inhibits hepatic glucose production by activating FOXO1, resulting in a reduction of hepatic glucose release. FOXO1 is a transcription factor that belongs to a subclass of the forkhead family of transcription factors that possess a forkhead box-type DNA binding domain. FOXO1 recognizes a specific regulatory element termed the insulin response element (IRE) on the promoters of glucose-6-phosphatase (G6Pase) and phosphoenolpyruvate carboxykinase (PEPCK) genes, both of which play important roles in maintaining glucose level in states of starvation [172,173,174]. Thus, through inhibition of FOXO1, insulin promotes glucose storage as glycogen and inhibits glucose synthesis and hepatic glucose output [175] (Figure 5).

Similar to the case in insulin-sensitive tissues, in states of IR, physiologic levels of circulating insulin are insufficient to elicit the appropriate insulin response in hepatic cells [176]. In the liver, IR impairs glycogen synthesis, fails to suppress glucose production, enhances lipogenesis, and increases the synthesis of proteins such as the proinflammatory CRP. In fact, the abnormal production of proinflammatory proteins such as adipocytokines and cytokines, combined with conditions such as oxidative stress, can lead to an inflammatory state responsible for altered insulin response by the liver [175].

## 6. T2DM Outcomes/Complications: Cardiovascular Risk

**Figure 6:**
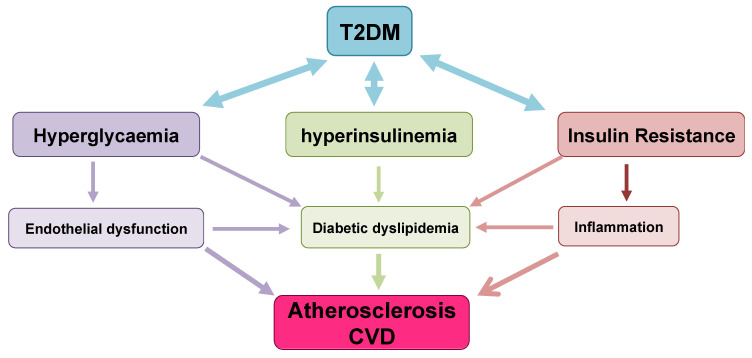
Factors implicated in cardiovascular risk outcomes from T2DM and the interactions between them. T2DM derived hyperglycemia, hyperinsulinemia and IR causes endothelial dysfunction, diabetic dyslipidemia and inflammation leading to CVD. The flowchart illustrates the multiple interactions among the implicated factors.

As described in the previous sections, T2DM is a multisystem disease with a strong correlation with CVD development [177]. T2DM leads to a two- to four-fold increase in the mortality rate of adults from heart disease and stroke and is associated with both micro- and macro-vascular complications, the latter consisting of accelerated atherosclerosis leading to severe peripheral vascular disease, premature coronary artery disease (CAD) and increased risk of cerebrovascular diseases [178,179,180]. These factors lead to T2DM being considered a significant risk factor for CVD [181], likely through the involvement of several molecular mechanisms and pathological pathways. These include the role of IR in atherosclerosis, vascular function, oxidative stress, hypertension, macrophage accumulation and inflammation [182,183,184,185]. The following sections describe in detail the main factors implicated in cardiovascular risk outcomes from T2DM and the interactions between them (Figure 6).

### 6.1. Diabetic Dyslipidaemia and Atherosclerosis Development

Dyslipidaemia is a common feature of T2DM, and increases the incidence of atherosclerosis and mortality of diabetic patients [186]. The hallmark of diabetic dyslipidemia is a characteristic dyslipidaemic profile consisting of elevated TG, TG-rich lipoproteins (TRLs), small dense LDLs (sdLDL), and reduced HDL levels [187,188,189]. Although the pathophysiology of dyslipidemia in T2DM is not completely characterized, several factors such as hyperglycemia, insulin resistance, hyperinsulinemia, abnormalities in adipokines and adipocytokines have been implicated [190]. Epidemiological studies indicate that TG-rich lipoproteins and their remnants contribute to atherogenesis and CVD risk [191,192,193,194,195] and experimental studies indicated a connection between cholesterol deposition and inflammation as a result of TRLs entry into the artery wall [196,197]. TRLs consist of a great variety of nascent and metabolically modified lipoprotein particles including intestine-derived apoB48 (chylomicrons and chylomicron remnants) and liver-derived apoB100 (VLDL and its remnants). TRLs are highly heterogeneous in size, density, and apolipoprotein composition [198].

**Figure 7:**
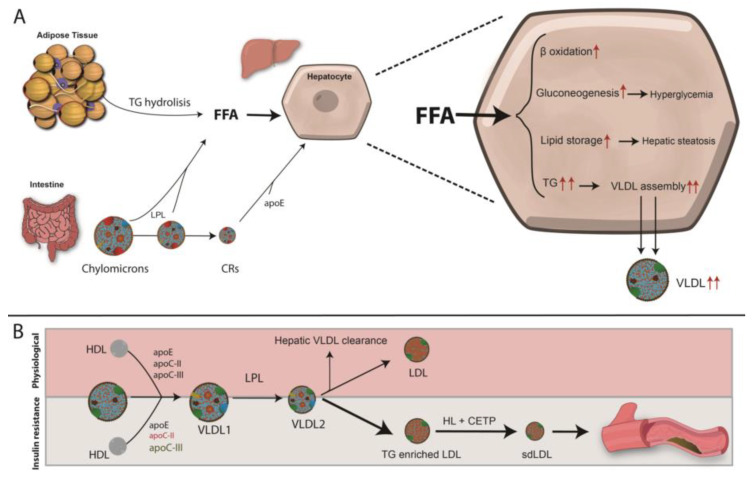
Diabetic dyslipidemia: mechanisms leading to T2DM dyslipidemia and lipoprotein clearance in physiological an IR conditions. (**A**) IR leads to an impaired adipose tissue fat storage, resulting in constitutive FFA release from the intracellular TG stores of adipocytes. The released FFAs are taken up by hepatocytes, where they can be directed to the mitochondria and undergo β-oxidation; be re-assimilated into TG to assemble new VLDL particles; shifted to gluconeogenesis resulting in a worsening of hyperglycemia; or stored as TG leading to hepatic steatosis. (**B**) Under physiological conditions, VLDL particles incorporate apo-CII and apoE from HDL allowing VLDL to be progressively lipolyzed leading to the generation of smaller VLDL particles (upper panel). T2DM and IR impair metabolism and clearance of chylomicrons and VLDLs. Activation of CETP promotes an exchange of TG out of RLPs and incorporates CE from HDL and LDL particles leading to reduced levels of circulating HDL-C and an increase in the more atherogenic sdLDL particles (lower panel). TG: triglyceride; FFA: free fatty acid, LPL: lipoprotein Lipase; CR: chylomicron remnants; HL: hepatic lipase; CETP: Cholesteryl Ester Transfer Protein; ApoE: apolopoprotein E; ApoC-II: apolipoprotein CII; apoC-III: apolipoprotein CIII; VLDL: very low-density lipoprotein; sdLDL: small dense lipoprotein.

Under physiological conditions, chylomicrons deliver dietary lipids and lipid-biliary sources to the liver, which upon entering central circulation acquire apoE, apo-CI, apo-CII and apo-CIII from circulating HDL (Figure 7). Apo-CII, an activator of lipoprotein lipase (LPL), hydrolyzes TG within the chylomicron core, thereby releasing free fatty acids (FFAs). The progressive removal of TGs leads to the formation of chylomicron remnants (CR), which upon apoE incorporation, are cleared by hepatocytes (Figure 7). This, together with the uptake of FFA generated by lipolysis in adipose tissue provides the major source of hepatic VLDL assembly and secretion. Once in the circulation, VLDL particles incorporate apo-CII and apoE from HDL allowing VLDL to be progressively lipolyzed leading to the generation of smaller VLDL particles (VLDL1, VLDL2 and VLDL3), IDL, and finally LDL (Figure 7). Lipoprotein production, metabolism, and clearance are efficient processes. However, T2DM and IR are among the most important metabolic derangements in these process and they give rise to impaired metabolism and clearance of chylomicrons and VLDLs [199,200].

#### Mechanisms Leading to T2DM Dyslipidemia and Atherosclerosis

Increased hepatic TG content present in T2DM patients leads to elevated hepatic production of VLDL and normal or slightly elevated LDL-C levels, most commonly sdLDLs enriched in TG [189,201]. One of the primary abnormalities in IR is impaired adipose tissue fat storage, resulting from insulin’s inability to inhibit hormone-sensitive lipase (HSL). This results in constitutive FFA release from the intracellular TG stores of adipocytes. The released FFAs are taken up by hepatocytes, where they can be directed to the mitochondria and undergo β-oxidation; be re-assimilated into TG to assemble new VLDL particles; shifted to gluconeogenesis resulting in a worsening of hyperglycemia; or stored as TG leading to hepatic steatosis.

The dominant feature of diabetic dyslipidemia is the increased production rate of VLDL-apoB100 by the liver, mainly VLDL1, which is related to insulin sensitivity indices [202]. This highlights the role of insulin on VLDL assembly and secretion by hepatocytes [203]. Insulin plays a role in almost all the steps of VLDL assembly and secretion. It is known that insulin inhibits the transcription of Mttp, the gene coding for the microsomal transfer protein (MTP), the protein responsible for assembling TG with apoB100 [204]. MTP facilitates concerted lipid transfer and apoB100 folding as it enters the ER lumen and lipidation determines the amount of the active pool of apoB100 [205]. Lipidation of apoB100 is a co-translational event and a rate-limiting step of apoB100 mRNA stability thus low availability of TG leads to apoB100 degradation. The addition of TG to apoB100 generates nascent VLDL particles that are transported to the GA by Sar2/COPII-containing vesicles. Within the GA, VLDL maturation occurs in a process promoted by the phospholipase D1 (PLD1) [203]. Therefore, in insulin-resistant condition, MTP expression and activity is increased thus contributing to raising apoB100 lipidation and to its rescue from degradation. Indeed, IR leads to a loss of the acute insulin-mediated inhibition of apoB100 secretion [205].

Availability of TGs within hepatocytes is important for VLDL synthesis [206] and the liver uses both de novo synthesized FAs and extra-hepatic FFAs as a substrate for TG synthesis [207]. De novo lipogenesis occurs primarily in the fed state in which the expression of lipogenic genes is regulated by the sterol regulatory element-binding protein (SREBP). The SREBP-1c isoform up-regulates almost all the enzymes involved in FA synthesis as well as enzymes that supply acetyl-CoA units and reducing equivalents to the pathway [208]. Insulin regulates SREBP-1c, which explains the lipogenic effect of chronic hyperinsulinemia [208]. FFAs derived from adipose tissue is also a major source of liver TGs and VLDL production. As mentioned above, T2DM is characterized by increased production of FFAs by adipose tissue [207]. Therefore, in IR, an increase in TG lipolysis in adipose tissue and FFA influx serves as another source of lipid to the liver [207].

As mentioned above, in the IR milieu, insulin has reduced capacity to inhibit VLDL secretion in the fed state, the availability of apo-CII is lower and apo-CIII production is increased [209]. These events result in the accumulation of VLDL remnants and IDL due to diminished clearance of TRLs by hepatocytes [210]. Additionally, hepatic IR also impairs LRP1 translocation from intracellular vesicles to the hepatocyte plasma membrane, which contributes to impaired clearance of TRLs [211,212] (Figure 7).

In an effort to offload TG from remnant lipoproteins (VLDL 2+3 and IDL; RLPs), CETP is activated and promotes an exchange of TG out of RLPs and incorporates CE from HDL and LDL particles [213]. The TG-enriched HDL and LDL particles are better substrates for lipolysis by hepatic lipase leading to reduced levels of circulating HDL-C and an increase in sdLDL particles, which are more atherogenic [214]. The increased movement of CE into circulating TRLs mediated by enhanced activity of CETP [215] plays a key role in generating small dense HDL and LDL particles, the former being less atheroprotective and the latter more atherogenic [216]. TG enrichment of HDL enhances circulating HDL clearance [217].

The lower HDL concentration and modified composition of HDL have an important impact in diminishing the particle capacity of inducing cholesterol efflux from the cells, which is the first step in reverse cholesterol transport (RCT) [218]. The impaired RCT activity has been associated with increased risk of CAD [219] and with flow-mediated vasodilation in T2DM patients [220].

Atherosclerosis is accelerated by the increased permeability of sdLDL into the subendothelial space (Figure 7) [221,222]. SdLDL particles are characterized by a lower affinity for LDLR due to conformational rearrangements occurring in apoB100 as the particle decreases in volume and size [223]. Furthermore, sdLDL particles are more susceptible to oxidation and thus avidly scavenged by activated macrophages in the subendothelial space, giving rise to foam cells [224]. In addition, sdLDL particles show increased proteoglycan binding and facilitated entry into the arterial wall, increased arterial retention, and a longer half-life [225]. sdLDL particles are also more likely to be glycated, more resistant to breakdown, and more susceptible to oxidation by free radicals [226].

There is an insulin response element in the gene for apoA-I, the primary apolipoprotein constituent of HDL particles [227,228]. As the liver becomes more insulin resistant, less apoA-I is produced and there is less HDL biogenesis. Adipocytes express the ATP-binding membrane cassette transport protein A1 (ABCA1). IR downregulates the expression of ABCA1 on the surface of adipocytes and reduces HDL formation by these cells [229,230,231]. Chylomicrons are enriched with apoA-I. IR reduces the release of this apoA-I into the serum by inhibiting LPL. In addition, within the milieu of IR of diabetes, HDL particle concentrations are not only quantitatively reduced, but also tend to be dysfunctional and unable to perform their primary functions, including reversal of cholesterol transport and inhibition of oxidative and inflammatory phenomena [232].

This highly atherogenic lipid profile is a pivotal contributor to atherogenic dyslipidemia, which is causally linked to the development and progression of atherosclerotic CV disease (ASCVD) [233,234]. The relationship between atherogenic dyslipidemia and ASCVD is supported by prospective longitudinal cohorts, clinical evidence and genetic linkage studies. As an example, the best predictor of risk of myocardial infarction at the population level in the INTERHEART study was the apolipoprotein (apo) B100/apoA-I ratio, reflecting the correlation between all apoB (atherogenic lipoproteins) and HDL (representing classically anti-atherogenic particles) [235]. The relationship between atherogenic dyslipidemia and ASCVD has also been demonstrated in prospective randomized clinical trials using statins. Even when treated with statins, patients with the atherogenic dyslipidemia phenotype have a higher risk of CV events than those without AD [236,237].

Diabetic dyslipidemia acts in concert with other metabolic and vascular abnormalities to further compound vascular risk. Chronic hyperglycemia induces endothelial dysfunction through a variety of mechanisms such as by reducing vasodilation, increasing vasoconstriction, increasing exposure to free radicals and impairing endothelial cell function, with a net effect of facilitating pro-atherogenic conditions [238]. Increased activity of the renin-angiotensin axis has also been found to further increase oxidative stress [239].

### 6.2. Impaired Endothelial Function and Atherosclerosis Development

Endothelium plays an important role in the regulation of vascular tone and structure through a balanced release of endothelial-derived relaxing and contracting factors. This balance is altered in T2DM leading to alteration of the physicochemical properties of the vascular wall via endothelial dysfunction, oxidative stress, platelet hyperreactivity, and inflammation [240,241]. These abnormalities lead to enhanced vasoconstriction, development of atherosclerosis, and favored thrombus formation [179,180].

#### 6.2.1. Mechanisms Leading to Endothelial Dysfunction in T2DM

Vascular endothelial cells are particularly susceptible to developing intracellular hyperglycemia because glucose diffuses passively through their plasma membrane. In T2DM, the excess of glucose can be metabolized in the sorbitol pathway to sorbitol and fructose by aldose reductase, which activates the aldose reductase secondary metabolic pathway, with concomitant oxidation of NADPH to NADP^+^ and reduction of NAD^+^ to NADH. NADPH depletion and an increased NADH/NAD^+^ cytosolic ratio leads to a change in redox potential that accelerates glycolysis and increases de novo synthesis of DAG [242]. As a result, protein kinase C (PKC) is activated, nitric oxide (NO) is reduced. These effects cause vascular permeability and increase contractility. Simultaneously, the increased NADH/NAD^+^ ratio also results in higher production of O_2_^−^, LDL oxidation, cytotoxic effects on endothelial cells and reduced NO availability, leading to endothelial dysfunction [241,242].

The overproduction of aldoses by the sorbitol pathway promotes protein glycosylation that yields the formation of the stable Amadori products (such as glycosylated hemoglobin) and AGEs. AGEs are associated with several molecules that augment oxidant activity and consequently the production of ROS, which increases oxidative stress and prevents the release of NO, resulting in vascular lesions. AGEs may also reduce endothelium-derived NO bioavailability and activity, further compromising vascular activity [243]. In addition, AGEs can trigger an inflammatory and pro-coagulant state and can cause endothelial activation through the induction of receptor-mediated gene transcription. AGE binding to the RAGE-receptor, nuclear transcription factor NF-κB [242,243] is activated leading to transcription of endothelin-1, VCAM-1, ICAM-1, E-selectin, thrombomodulin, TF, vascular endothelial growth factor (VEGF), IL-1, IL-6, and TNF-α [242,244]. Increased expression of inflammatory and adhesion molecules amplifies the inflammatory response and aggravates diabetic vascular complications. These pro-inflammatory cytokines stimulate the expression and release of pro-coagulant molecules and inhibit the expression of anti-coagulant molecules by endothelial cells [245]. This leads to a pro-coagulant state in the surface of the endothelium and increases growth factor production resulting in a thickening of the basement membrane, thus favoring protein and lipid deposition and impairing vasodilation [242,246].

#### 6.2.2. Endothelial Dysfunction in T2DM and Atherosclerosis Development

Hyperglycemia-associated vascular injury, oxidative stress, inflammation and altered hemodynamic balance may initiate atherosclerosis development and formation of arterial thrombus [247]. At early stages of atherosclerosis, circulating LDL binds to matrix proteoglycans where their oxidation is favored, giving rise to highly pro-inflammatory particles that stimulate the expression of several adhesion molecules by endothelial cells [242,248]. This promotes selective binding of leukocytes and their transmigration into the vascular wall along with recruitment and activation of circulating monocytes that differentiate into macrophages. The excess of oxidized LDL is removed by macrophages by a non-regulated mechanism that leads to the formation of foam cells and the onset of fatty streaks. Mononuclear cells release inflammatory cytokines, including IL-1 and IL-6, promoting the recruitment of additional inflammatory cells. As a result, smooth muscle cells proliferate and migrate into the intima where they synthesize and secrete extracellular matrix facilitating fibroatheroma formation [242]. As the process progresses, if a fissure or ulceration of the plaque occurs, highly thrombogenic substances are exposed leading to adhesion and aggregation of platelets, which promotes thrombus formation [249]. In addition, platelets can also release pro-inflammatory cytokines and growth factors promoting monocyte recruitment to atherosclerotic plaques, which stimulates fibroblasts and smooth muscle cell proliferation thus accelerating the atherosclerotic process.

### 6.3. Diabetes-Associated Chronic Inflammation and Atherosclerosis Progression

A critical component of T2DM is a chronic low-grade inflammatory state, referred to as “metaflammation” [250]. This chronic condition involves the same cellular and molecular players of acute inflammatory responses and has been suggested as an underlying cause of the progression of atherosclerosis in T2DM. Hyperglycaemia can increase circulating cytokines that can lead to chronic inflammation in T2DM [250]. Among them, patients with T2DM have higher levels of IL-1b, IL-6, IL-8, MCP-1, and other major cytokines in both monocytes and macrophages [130]. The underlying mechanisms involved in this process are ROS-mediated activation of p38 and other proinflammatory kinases, upregulation of NF-kB induction, oxidative stress, and activation of the AGE-RAGE pathway [129,130]. In addition, exposure to high glucose levels impairs the phagocytic activity of macrophages, which partially explains the increased incidence of chronic infection among T2DM patients [251]. Indeed, T2DM is associated with increased activity of the inflammasome, upregulation of the nucleotide-binding oligomerization domain-like receptor 3 (NLRP3), increased levels of IL-1β and IL-18 [252,253,254]. These events trigger neutrophil extracellular trap activation, or NETosis, a characteristic cell death of macrophages causing chronic inflammation [255]. High levels of these markers have been found in T2DM patients [256], which are enhanced in hyperglycaemic conditions [257].

### 6.4. Adipokine Balance and CVD Risk

Adipose tissue dysfunction as a result of T2DM can result in an imbalance between pro-inflammatory and anti-inflammatory adipokines, and is one of the mechanisms of T2DM complications. Several studies indicate that adipokines are related to IR, and can result in endothelial dysfunction, and pro-inflammatory and pro-atherogenic states [258,259].

Adiponectin is a well-described insulin-sensitizing hormone and its expression and circulating levels are inversely proportional to the extent of adiposity. Adiponectin has insulin-sensitizing properties [260,261]. Adiponectin acts through ADIPOR1 and ADIPOR2 receptors [262] and the peroxisome proliferator-activated receptor α (PPARα) pathway, leading to decreased hepatic gluconeogenesis, increased liver and skeletal muscle fatty acid oxidation, increased glucose uptake in skeletal muscle and white adipose tissue, and decreased white adipose tissue inflammation [263]. In addition, adiponectin ameliorates β-cell death by neutralizing inflammatory and lipotoxic ceramides and DAGs [264] and shows strong anti-inflammatory effects on other cell types such as macrophages and fibrogenic cells [263,265,266]. Low concentrations of adiponectin have been found in T2DM patients and are correlated with increased risk of developing premature arteriosclerosis, and are thus considered an additional CVD risk factor [267]. Notably, adiponectin deficiency is associated with coronary artery disease, hypertension, endothelial dysfunction and greater carotid intima-media thickness [268,269,270,271]. Low concentrations of adiponectin lead to an increased expression of intercellular adhesion molecule-1 (ICAM-1), vascular cell adhesion molecule-1 (VCAM-1) and E-selectin, promotes differentiation of macrophages into foam cells and enhances the proliferation and migration of smooth muscle cells [272].

Omentin is an adipokine secreted from white adipose tissue and is involved in glucose homeostasis [273,274]. Omentin circulates in the blood [275,276], and is associated with reduced levels in T2DM patients [277,278]. In vitro studies have shown that omentin enhances insulin-stimulated glucose uptake in human adipocytes by AKT signaling pathway activation [273]. In humans, an inverse correlation between omentin levels and IR is seen, both at the protein and mRNA levels [277,279,280]. Additional studies show that omentin has anti-inflammatory properties, diminishes cytokine expression [281,282], and is negatively associated with systemic inflammatory markers such as TNF and IL-6 [283].

Vaspin (visceral adipose tissue-derived serine protease inhibitor) is an adipokine that inhibits proteases responsible for IR and protects against atherosclerosis and plaque development [284,285]. It has been shown that T2DM patients have higher serum vaspin levels than healthy controls. Higher vaspin levels are associated with a 1.7-fold increased risk of CVD [70]. High vaspin is also associated with increased severity of coronary artery disease [286].

## 7. Conclusions

The importance of research in the fields of glucose homeostasis, insulin and diabetes has not faded. In fact, due to rapid globalization and the normalization of a sedentary lifestyle, along with increased obesity, diabetes and their consequent co-morbidities, research in this topic must continue to grow. Understanding the mechanisms implicated in every step in the development and complications of T2DM is crucial in order to prevent, control, treat or revert the pathophysiology of T2DM its complications. Although quality outcomes for patients are optimized by early detection of T2DM through screening and intensive patient-centered management, research efforts are needed to define causative factors accounting for correlations among different demographic subsets and the corresponding variable risks for T2DM as well as the drivers of increased risk in individuals of low socioeconomic status. Being the pathophysiology and underlying mechanisms of T2DM increasingly understood, precision medicine should be implemented and treatments individualized and targeted appropriately with the help of molecular genetic tools by identifying specific variants contributing to disease development as well as by searching biomarkers to assess progression and response to therapeutic interventions. Additional research is needed to determine a direct causal role of the intestinal microbiota in pathogenesis of T2DM and response to therapies needs to be determined.

Taking everything in this review into consideration, it is clear that there is still a long way until we fully understand each of the many stakeholders in glucose homeostasis.
